# Influence of organization and demographic characteristics of primary care practices on continuity of care: analysis of a retrospective cohort from 287 primary care practices covering about 6 million people in Catalonia

**DOI:** 10.1186/s12875-021-01414-y

**Published:** 2021-03-25

**Authors:** Ermengol Coma, Núria Mora, Paula Peremiquel-Trillas, Mència Benítez, Leonardo Méndez, Albert Mercadé, Francesc Fina, Mireia Fàbregas, Manuel Medina

**Affiliations:** 1 Sistemes d’Informació dels Serveis d’Atenció Primària (SISAP), Institut Català de la Salut (ICS), Gran Via de Les Corts Catalanes, 587, 08007 Barcelona, Spain; 2 Fundació Institut Universitari per a la Recerca a l’Atenció Primària de Salut Jordi Gol i Gurina (IDIAPJGol), Barcelona, Spain; 3 Preventive Medicine and Epidemiology Department, Hospital Universitari Vall d’Hebron, Barcelona, Spain; 4 Unit of Molecular Epidemiology and Genetics in Infections and Cancer, IDIBELL, Catalan Institute of Oncology, L’Hospitalet de Llobregat, Barcelona, Spain; 5 Equip d’Atenció Primària Gòtic, Institut Català de La Salut (ICS), Barcelona, Spain

**Keywords:** Continuity of patient care, Primary health care, Healthcare services utilization, Patient care management, Health service research, Patient-physician relationship, Population health management

## Abstract

**Background:**

There is evidence that an ongoing patient-physician relationship is associated with improved health outcomes and more efficient health systems. The main objective of this study is to describe the continuity of care in primary healthcare in Catalonia (Spain) and to analyze whether the organization of primary care practices (PCP) or their patients’ sociodemographic characteristics play a role in its continuity of care.

**Methods:**

Four indices were used to measure continuity of care: Usual Provider Index (UPC), Modified Modified Continuity Index (MMCI), Continuity of Care Index (COC), and Sequential Continuity Index (SECON). The study was conducted on 287 PCP of the Catalan Institute of Health (Institut Català de la Salut—ICS). Each continuity of care index was calculated at the patient level (3.2 million patients and 35.5 million visits) and then aggregated at the PCP level. We adjusted linear regression models for each continuity index studied, considering the result of the index as an independent variable and demographic and organizational characteristics of the PCP as explanatory variables. Pearson correlation tests were used to compare the four continuity of care indices.

**Results:**

Indices’ results were: UPC: 70,5%; MMCI: 73%; COC: 53,7%; SECON: 60,5%. The continuity of care indices had the highest bivariate correlation with the percentage of appointments booked with an assigned health provider (VISUBA variable: the lower the value, the higher the visits without an assigned health provider, and thus an organization favoring immediate consultation). Its R^2^ ranged between 56 and 63%, depending on the index. The multivariate model which explained better the variability of continuity of care indices (from 49 to 56%) included the variables VISUBA and rurality with a direct relationship; while the variables primary care physician leave days and training practices showed an inverse relationship.

**Conclusion:**

Study results suggest that an organization of primary care favoring immediate consultation is related to a lower continuity of patient care.

**Supplementary Information:**

The online version contains supplementary material available at 10.1186/s12875-021-01414-y.

## Background

Continuity of care has been defined as a personal long-standing relationship between a patient and a single doctor [1]. This relationship implies seeing each other repeatedly and thus establishing a long-lasting personal relationship based on stability, empathy, and trust [2, 3]. Continuity of care is a proxy measure of patient-doctor relationship strength [4], as it creates a bond between patients and doctors (“my doctor”, “my patients”) [1].

There is evidence that continuity of care is associated with increased health system efficiency [5], increased patient satisfaction, improved patient-doctor relationships [3] and reduced use of emergency care, and fewer hospital admissions [5–7]. It is also related to a better quality of care, including improved diagnosis precision [8], enhanced medical adherence [9], improved control of chronic diseases, benefits in preventive medicine, and reduced patient mortality [10, 11].

Therefore, continuity of care should be a top priority for primary care practices [9, 10, 12]. Recent articles, however, have pointed out some threats to the continuity of care related to organizational issues like precarious working conditions and understaffing, segmentation of primary healthcare activities and the prioritization of access over continuity, which creates an increase of immediate attention [12, 13]. This last threat is usually a consequence of the increase in waiting time for anything other than an emergency [13]. Despite the increase in organizational models in primary care practices (PCP) that promote immediate consultation to improve access (regardless of the real urgency of the medical problem) [10], there is no literature providing quantitative evidence of its effect on continuity of care.

The Catalan Institute of Health (Institut Català de la Salut—ICS) is the main provider of primary care services in Catalonia and its 287 PCPs cover about 6 million people (80% of the population in Catalonia). The Catalan Health Service is the public agency that guarantees the right of health care for all citizens in Catalonia through a health insurance funded by state taxes. Every citizen is assigned to a specific doctor and nurse, which form a basic healthcare unit. This healthcare unit acts as a team and provides care to the same group of patients [14]. Usually a patient couldn’t go to any PCP but the one that corresponds according to the place of residence. However patients could change this assignment and choose which doctor or nurse they prefer to be assigned to. For example, if a patient changes his place of residence he still can keep his doctor and doesn’t need to change. But after this decision is done, when a patient books an appointment he only can book for his specific doctor or nurse. Despite the majority of practices using this structure, there is a growing trend of PCP providing immediate consultation through "emergency" visits that disregard the patient's specific doctor assignment [12].

In this study, our aim was to describe the continuity of care in ICS's primary care practices and to analyze whether an organization favoring immediate consultation has an effect on continuity.

## Methods

### Study design details

We performed a retrospective cohort study in all 287 PCP of Institut Català de la Salut (ICS) in Catalonia, Spain. These PCP have an adult (age ≥ 15 years) assigned population of 4.912.432. Three PCP were excluded due to the unavailability of some variables.

To measure continuity of care we included all adult patients with 3 or more visits with a primary care physician (GP) in a period of two years (December 2017 to November 2019).

### Main variable

The main variable was continuity of care in PCP level. For its measurement, we used four international indices identified through bibliographic search [15–19]:

- Usual Provider of Care index (UPC): Measures the proportion of visits performed by the GP that the patient visited most frequently out of all visits.
- Modified Modified Continuity index (MMCI): Measures the number of GPs providing healthcare to a patient and the proportion provided by each one over a period of time. This index focuses on the dispersion between GPs and is based on the number of GP and number of visits only.
- Continuity of Care index (COC): Measures continuity of care based on frequency and dispersion of visits between GPs. This index combines aspects of UPC and MMCI.
- Sequential Continuity Index (SECON): Measures the proportion of sequential visits made by the same GP over a period of time.

Formulae and examples for every index are detailed in an additional file (see Additional file 1).

Firstly, we measured the continuity of care for every individual patient. Then, we aggregated the measure at the PCP level with the average continuity of those assigned to a PCP. This aggregation attempted to avoid the effect of patients with an extreme number of visits (hyperfrequentative patients) on the aggregated measurement of continuity of care.

### Explanatory variable

Socio-demographic variables were aggregated for each PCP. They included the patient's mean age, gender (percentage of women), percentage of immigration from a low-income country, and mean morbidity of the PCP measured through the complexity index of the adjusted morbidity groups (AMG) [20, 21].

Rural areas were defined as areas with less than 10,000 inhabitants and a population density lower than 150 inhabitants/km^2^. We assessed socioeconomic status using the validated socioeconomic index (ISC) from Catalan Agency for Healthcare Quality and Assessment (Agència de Qualitat i Avaluació Sanitàries de Catalunya, AQuAS), calculated at the PCP level [22].

We also included some other PCP variables, such as being a GP training practice, accessibility (measured as the percentage of assigned patients who would get an appointment with their respective GP in less than 48 h), and mean number of GP leave days. These leaves included the number of days of maternity, paternity and sick leaves for every GP from a PCP divided by the number of GP working in the PCP. Training practices services were considered as those training intern GP [23].

Finally, an organizational variable to assess the organizations’ orientation towards immediacy of the GP consultations was included (VISUBA variable). In the Catalan health system, when patients demand for consultation, an appointment is booked on a schedule. These schedules may be managed by a single GP (an assigned GP) or by any GP in the PCP (schedules used for emergency consultation where patients do not know beforehand which GP is going to visit them). Each PCP can decide the kind of schedule distribution to use. The VISUBA variable was firstly proposed as an organizational variable at the biggest primary care scientific meeting in Spain [24] as the percentage of visits made in schedules managed by a single-GP out of the total number of visits in the PCP. VISUBA variable is thus a continuous scale, where the lower the value the higher the degree of immediate consultation. We deemed PCP favoring immediate consultation, those with VISUBA lower than 80% [24].

### Information sources

Data used to measure continuity indices, socio-demographic and organizational variables were obtained from anonymized primary care electronic medical records (EMR) of Institut Català de la Salut (ICS). ICS is the main primary health care provider in Catalonia and manages about 4 in every 5 practices in the region, all using the same EMR software, named ECAP [25].

For this particular study, EMR data were linked with ICS's human resources (HR) department in order to measure the GP leaves days and with data from AQuAS for the socioeconomic deprivation index [22].

### Statistical analysis

Numerical variables were described with the mean and median for central tendency; and standard deviation, quartiles, maximum, and minimum values were used to analyze dispersion.

For categorical variables, the absolute and the relative frequency of each category was calculated.

To assess correlation for the four continuity indices, a correlation matrix using Pearson correlation coefficient was measured.

Bivariate analyses for numerical variables were performed using Pearson correlation coefficient. Analyses of categorical and numerical values were performed through T-Student method.

A linear regression model using the continuity of care as the main dependent variable was adjusted for every index. The explanatory variables that have been included in the model as adjustment variables were those that have obtained a p-value of less than 0.1 in the previous bivariate test. Adjustment variables with a p-value lower than 0.05 in Wald test were considered significant.

All statistical analyses were conducted using R software, version 3.5.1 [26].

## Results

Overall, we included 3.199.185 patients older than 14 years with 3 or more clinical visits in primary care in the last two years (a total of 35.478.718 visits) to calculate the continuity of care indices.

Table 1 summarizes the characteristics of the PCP included in our study. 65% of the PCP analyzed were urban and close to 25% were GP training practices.

**Table 1:** Baseline characteristics of all the PCP included in the study. Variables are presented with mean (standard deviation)

**Variables**	**Total (*N* = 284)**	**Rural (*N* = 99)**	**Urban (*N* = 185)**	***P*-value**
Mean age of patients (years)	48.9 (2)	49.9 (2.1)	48.4 (1.7)	0.00
Population assigned to practices	17,141 (7,629)	11,853 (7,263)	19,971 (6,190)	0.00
% of women	50.8 (2)	49.6 (1.3	51.4 (2)	0.00
% of immigration from a low-income country	12.7 (7)	11.3 (5.8)	13.5 (7.5)	0.01
Mean morbidity (AMG complexity index)	4.5 (0.5)	4.5 (0.4)	4.5 (0.5)	0.35
Socioeconomic deprivation index	47.6 (15.7)	47.3 (9.9)	47.7 (18)	0.83
Accessibility	31 (20.4)	46.1 (25.3)	22.9 (10.5)	0.00
VISUBA (Percentage of appointments booked with an assigned GP)	87.9 (9.9)	92.7 (7)	85.3 (10.2)	0.00
Leave days per GP (last 2 years)	40.8 (29)	40.1 (32.5)	41.2 (26.9)	0.77
Number and percentage of training practices with resident intern GP	68 (23.9%)	15 (15.2%)	53 (28.7%)	0.02

The four continuity indices were measured for every PCP (Table 2). Mean values for each index were:

- UPC: 70.51% ± 6.76%
- MMCI: 73.03% ± 6.61%
- COC: 53.67% ± 9.2%
- SECON: 60.53% ± 8.59%

**Table 2:** Description of continuity of care indices for PCP

	**Mean (SD)**	**Minimum**	**Maximun**
**UPC**	70.51% (6.76)	47.27%	90.81%
**MMCI**	73.03% (6.61)	52.56%	91.65%
**COC**	53.67% (9.2)	25.25%	83.51%
**SECON**	60.53% (8.59)	34.58%	85.6%

Median values for the indices ranged from 53.79% to 73.91%.

**Fig. 1:**
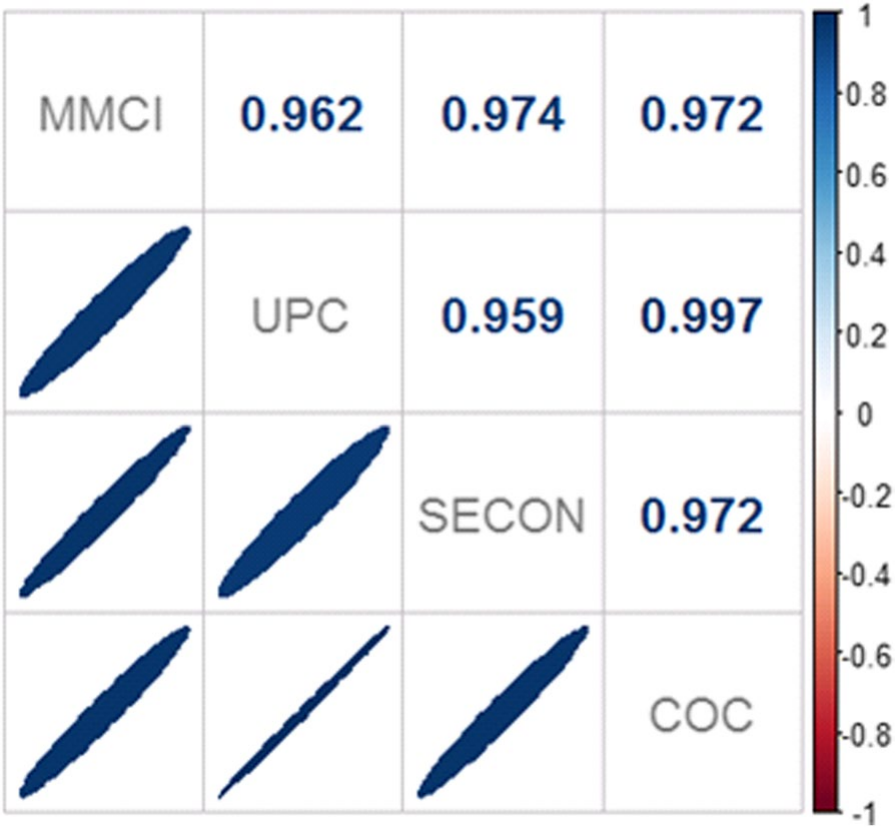
Correlations between continuity of care indices at PCP level. Legend: UPC: Usual Provider Index; MMCI: Modified Modified Continuity Index; COC: Continuity of Care Index; SECON: Sequential Continuity Index

A high correlation among indices was found (Fig. 1), with values ranging from 0.959 to 0.997.

A bivariate analysis using PCP characteristics showed a higher continuity of care in rural practices, non-training practices and PCP without immediate consultation (Table 3).

**Table 3:** Results of continuity of care indices according to PCP characteristics (categorical variables)

	**UPC**	**MMCI**	**COC**	**SECON**
	**Mean (SD)**	**Mean (SD)**	**Mean (SD)**	**Mean (SD)**
Rurality				
Rural	73.89% (6.4)	77.41% (5.5)	58.50% (8.8)	65.52% (7.5)
Urban	68.71% (6.3)	70.68% (5.9)	51.08% (8.3)	57.85% (7.9)
*P value*	< 0.0001	< 0.0001	< 0.0001	< 0.0001
Immediate consultation organization (VISUBA < 80%)				
No	72.81% (5.8)	75.56% (5.5)	56.85% (8.0)	63.88% (7.01)
Yes	66.01% (6.2)	68.06% (5.8)	47.43% (8.1)	53.97% (7.6)
*P value*	< 0.0001	< 0.0001	< 0.0001	< 0.0001
Training practice				
No	71.92% (6.2)	74.32% (6.2)	55.62% (8.6)	62.48% (7.8)
Yes	66.04% (6.2)	68.92% (6.1)	47.47% (8.2)	54.31% (8.0)
*P value*	< 0.0001	< 0.0001	< 0.0001	< 0.0001

Table 4 presents the correlations of continuity of care indices and numerical explanatory variables. The variable with the highest correlation was also VISUBA, measured as a continuous variable, with an R^2^ value of 0.63 on SECON index, and with high values on the other indices (0.56 UPC, 0.57 COC y 0.62 MMCI). Significant, though minor, statistical correlations, were also found between continuity indices and accessibility, GP leave days, socioeconomic deprivation, percentage of immigration from a low-income country, median age of the population and PCP population size (Table 4).

**Table 4:** Correlations^a^ between continuity of care indices and continuous variables of the PCP

**Variables**	**UPC**	**MMCI**	**COC**	**SECON**
VISUBA	0.56^a^	0.62^a^	0.57^a^	0.63^a^
Accessibility < 48 h	0.34^a^	0.45^a^	0.36^a^	0.37^a^
GP leave days (2 year period)	-0.29^a^	-0.27^a^	-0.27^a^	-0.2^a^
Socioeconomic deprivation index (AQUAS)	-0.23^a^	-0.16^a^	-0.21^a^	-0.2^a^
Median age	0.29^a^	0.35^a^	0.3^a^	0.33^a^
Adjusted morbidity (GMA)	-0.08	-0.02	-0.07	-0.07
% of immigration from a low-income country	-0.14^a^	-0.14^a^	-0.14^a^	-0.15^a^
% women patients	-0.04	-0.16^a^	-0.07	-0.11
PCP practice size	-0.18^a^	-0.33^a^	-0.21^a^	-0.24^a^

^a^ Correlations between continuity of care indices and variables are statistically significant

Linear regression models were measured including variables that showed a significant statistical correlation with continuity of care. The model with the highest explanatory power included continuous VISUBA, GP leave days, GP training practice, and rurality. Table 5 presents each model’s coefficients and R^2^ values. These ranged from 56 to 49% depending on which continuity index was assessed. To assess the influence of the VISUBA variable, a linear regression model without other variables was measured. An R^2^ value close to 40% was found for MMCI and SECON indices.

**Table 5:** Selected models and beta coefficients with 95% confidence interval and *R*^2^ value

**Variables**	**UPC (IC95%)**	**MMCI (IC95%)**	**COC (IC95%)**	**SECON (IC95%)**
(Intercept)	50.09 (43.92; 56.26)	52.06 (46.50; 57.62)	28.39 (21.10; 35.68)	25.62 (17.28; 33.97)
VISUBA	0.29 (0.22; 0.35)	0.30 (0.24; 0.36)	0.40 (0.31; 0.48)	0.43 (0.36; 0.50)
GP leave days(2 year period)	-0.06 (-0.08; -0.04)	-0.05 (-0.07; -0.03)	-0.08 (-0.10; -0.05)	-0.04 (-0.07; -0.02)
GP training practices	-4.87 (-6.23; -3.50)	-4.00 (-5.23; -2.78)	-6.66 (-8.50; -4.81)	-6.42 (-8.03; -4.81)
Urban practices	-2.35 (-3.64; -1.05)	-3.96 (-5.13; -2.80)	-3.55 (-5.29; -1.80)	-3.61 (-5.14; -2.08)
**Adjusted model** ***R***^**2**^	49%	56%	49%	56%
**VISUBA only adjusted model** ***R***^**2**^	32%	39%	33%	40%

## Discussion

To our knowledge, this is the first study measuring the effect of PCP characteristics and their organizational structures on continuity of care. PCP which favored immediate consultation showed lower continuity of care with differences ranging from 6 to 9%. These results are useful to quantify the previously stated idea that an organizational model favoring immediate consultation in primary care had a negative effect on continuity of care [12]. Furthermore, this loss of continuity occurred at every level of the immediate consultation organizations.

GP maternity, paternity leaves as well as annual sick leave days entailing a negative effect on continuity of care is an expected result although to our knowledge are scarcely published in literature. Rural PCP showed higher values on every continuity index. MMCI index had the highest difference, with a 6.7% higher value on rural practices. Despite being stated in literature before [5], there are no studies with an explanation for this effect no an analysis between rurality, continuity of care and organizational models.

Our study also found that training practices (those with intern resident GP) had an UPC value of 66%, contrasting to a 72% in non-training practices. From a theoretical standpoint, this decrease in continuity of care is expected, because GP and intern resident are two different doctors attending the same patient populations, causing a negative effect on the measurements of all indices. However, some studies talk about "continuity of supervision". This concept is defined as a collaborative health service provision conducted by a team of two GP working with the same patients, taking consensual or supervised decisions [27–29]. Considering formative experience as an essential part of the future and present of primary care, further studies on this matter are needed to really assess whether this lower continuity of care due to continuity of supervision at the PCP level has an effect on health outcomes.

Continuity indexes in the Catalan primary care were higher than others described in the literature. Mean values ranged from 53 to 73%, thus being higher than results published by Gill et al. [30], where MMCI index values ranged from 48 to 51%. Barker et al. measured an UPC index of 61% [7], and Sidaway-Lee et al. found the same result [31], while in our study the mean value for UPC was 70.5% (SD 6,8). These differences may be due to the patient assignment model in Catalonia, which is a main difference with practices analyzed elsewhere. However, our results were lower than Dreiher et al. found in Israel, where similarly to our setting, patients are also assigned to a specific GP. Dreiher et al. found median values of UPC 76%, MMCI 81%, COC 67% and SECON 70% [15], yet those were measured with a much smaller cohort than ours and were not aggregated on a PCP level.

Among the limitations of our study, firstly we have to mention that possibly some unknown organizational factors and factors without available data have an impact on the continuity of care and influence patients-GP relationships. To address this limitation, we have included in the analysis all the available data covering the main aspects related to continuity of care at a PCP level, such as age, socioeconomic status, training practice, variables related to booking visits as accessibility, etc. Also, the study uses a variable to measure immediate consultation which allows the assessment of a relevant organizational model [12, 13]. Secondly, it is known that the characteristics of the population assigned to PCP affect the continuity of care [16]. This study showed a correlation between the continuity of care and socioeconomic deprivation (lower continuity of care in higher deprivation areas), as well as the percentage of the migrant population (lower continuity of care in a higher percentage of immigration from a low-income country) and the median age of the population (higher continuity of care in higher age). Even though these variables were aggregated on a PCP level and were not significant on the regression model, this does not mean that they don't have any effect on the continuity of care. It would be interesting to combine PCP and patient defining characteristics in a multi-level analysis to study all the different factors. This approach, however, goes further than the current goals of the present study.

Despite the limitations, this study also has strengths. The data used were obtained directly from primary-care records and are of good quality. Several studies have used the Catalan EMR to do useful research in real-world conditions [32–35]. We also measured four continuity of care indices with data from more than 3 million people and 35 million visits. These sample sizes are much higher than in any other previous study [3]. Besides, four continuity indices in our study were highly correlated (values ranged between 0.96 and 0.99) and this is consistent with evidence published in the literature [5, 16, 18]. Finally, a majority of previous studies focused on the effect of continuity of care on health results. Nonetheless, this study aims to generate knowledge from a different approach: the identifying of variables which have an effect on patients’ continuity of care in primary care practices.

## Conclusions

The results of our study suggest that a primary care organization favoring immediate consultation relates to a lower continuity of care. This is a key piece of information, useful when a primary care practice defines its organization model. Considering the known benefits of continuity in healthcare, such as a decrease in mortality, this loss must be accounted for when choosing an organization favoring immediate consultation.

## Supplementary Information

**Additional file 1.** Formulae for the computing of continuity of care indices and examples.

## Data Availability

The datasets used and/or analyzed during the current study are available from the corresponding author on reasonable request.

## Abbreviations

AMG: Adjusted morbidity groups
COC: Continuity of Care Index
ECAP: Electronic medical records software in Catalonia
EMR: Electronic medical records
GP: Primary care physician
ICS: Institut Català de la Salut
HR: Human resources
MMCI: Modified Modified Continuity Index
PCP: Primary care practices
SECON: Sequential Continuity Index
UPC: Usual Provider Index
VISUBA: Percentage of visits appointed in the assigned GP's schedule
