# Supplementary material for: Influence of organization and demographic characteristics of primary care practices on continuity of care: analysis of a retrospective cohort from 287 primary care practices covering about 6 million people in Catalonia
Source: BMC Fam Pract. 2021 Mar 25;22:56. doi: 10.1186/s12875-021-01414-y (PMC7992318; doi:10.1186/s12875-021-01414-y)
Supplement: Supplementary file 1 — Additional file 1. Formulae for the computing of continuity of care indices and examples. [file 12875_2021_1414_MOESM1_ESM.docx]

**Additional file 1**

**Formulae for the computing of continuity of care indices and examples**

1. ***Usual Provider of Care index - UPC*.**

**UPC = n_i_/N**

n_i_ = number of visits to the main GP by patient i

N = total number of patient i’s visits to a GP over a period of time

Example: We have two patients (P1 and P2) with this sequence of visits with two GPs (A and B) in the last two years:


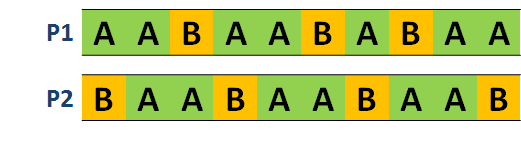


Which patient has a better continuity of care?

P1 has 7 visits with GP A and 3 with B. The UPC index is 0,7 (ni=7; N=10, UPC=7/10), a 70%

P2 has 6 visits with GP A and 4 with GP B. The UPC index is 60%.

1. ***Modified Modified Continuity Index - MMCI***

**MMCI = (1- (k/N+0,1)) / (1-(1/N+0,1))**

k = number of GPs.

N = number of visits by all GPs in a period of time.

Example: We have two patients (P1 and P2) with this sequence of visits with several GPs (A, B, C, D and E) in the last two years:


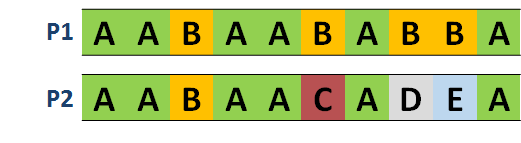


Which patient has a better continuity of care?

P1 has a MMCI value of (1-2/10,1)/1-1/10.1) = 0,89 (89%)

P2 has MMCI value of (1-5/10,1)/1-1/10,1) = 0,56 (56%)

Although both patients have the same UPC (60%).

1. ***Continuity of Care index - COC*.**

**COC = (Sum of squaring number of visits of each GP) - N /(N(N-1)**

N = number of visits by all GPs in a period of time.

Example: We have two patients (P1 and P2) with this sequence of visits three GPs (A, B and C) in the last two years:


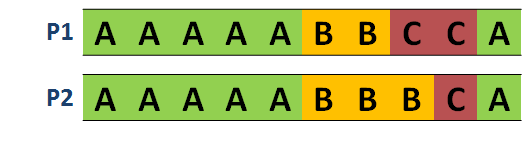


Which patient has a better continuity of care?

P1 has a COC value of 0,38 (38%) and P2 of 0,4 (40%), although both have the same UPC (60%) and MMCI (78%).

1. ***Sequential Continuity Index - SECON***

**SECON = t_i_ + … + t_n-1_ / N -1**

t = has a value of 1 if the current and next visits are made by the same GP, and a value of 0 if otherwise. The last visit of the time period is not accounted for..

N = number of visits by all GPs in a period of time.

Example: We have two patients (P1 and P2) with this sequence of visits with primary two GPs (A and B) in the last two years:


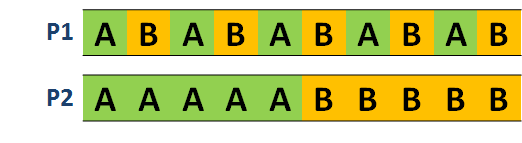


Which patient has a better continuity of care?

P1 has a SECON value of 0 and P2 of 0,89 (89%), despite both having the same UPC (50%), MMCI (89%) and COC (44%).
